# A thermophilic microorganism from Deception Island, Antarctica with a thermostable glutamate dehydrogenase activity

**DOI:** 10.1186/s40659-018-0206-3

**Published:** 2018-12-08

**Authors:** Patricio A. M. Flores, Daniela N. Correa-Llantén, Jenny M. Blamey

**Affiliations:** 1 Fundación Científica y Cultural Biociencia, José Domingo Cañas 2280, Santiago, 7750132 Chile; 2 Facultad de Química y Biología, Universidad de Santiago de Chile, Alameda 3363, Estación Central, Santiago, Chile

**Keywords:** Bacteria, *Bacillus*, Glutamate dehydrogenase, Biotechnology

## Abstract

**Background:**

The Antarctic continent is a source of extreme microorganisms. Millions of years of isolation have produced unique biodiversity with adaptive responses to its extreme environment. Although the Antarctic climate is mainly cold, the presence of several geothermal sites, including thermal springs, fumaroles, hot soils and hydrothermal vents, provides ideal environments for the development of thermophilic and hyperthermophilic microorganisms. Their enzymes, called thermoenzymes, are the focus of interest in both academic and industrial research, mainly due to their high thermal activity and stability. Glutamate dehydrogenase, is an enzyme that plays a key role in the metabolism of carbon and nitrogen catalyzing reversibly the oxidative deamination of glutamate to alpha-ketoglutarate and ammonium. It belongs to the family of oxidoreductases, is widely distributed and it has been highly regarded for use as biosensors, particularly for their specificity and ability to operate in photochemical and electrochemical systems. However, the use of enzymes as biosensors is relatively problematic due to their instability to high temperatures, organic solvents and denaturing agents. The purpose of this study is to present the partial characterization of a thermophilic microorganism isolated from Deception Island, Antarctica, that displays glutamate dehydrogenase activity.

**Results:**

In this work, we report the isolation of a thermophilic microorganism called PID15 from samples of Deception Island collected during the Antarctic Scientific Expedition ECA 46. This microorganism is a thermophile that grows optimally at 50 °C and pH 8.0. Scanning electron microscopy shows rod cells of 2.0 to 8.0 µm of length. Phylogenetic analysis of 16S rRNA gene revealed that this microorganism is closely related to *Bacillus gelatini*. This microorganism contains a thermostable glutamate dehydrogenase with optimal activity at pH 8.0 and temperatures for its activity from 37 to 50 °C, range of temperature of interest for biotechnological applications. This glutamate dehydrogenase is a highly thermostable enzyme.

**Conclusion:**

This is the first report of a microorganism from Antarctica containing a thermostable glutamate dehydrogenase that maintains its activity in a broad range of temperatures making it of potential interest for biotechnological applications.

## Introduction

Environmental conditions in Antarctica are different from all other places of the planet. Although the Antarctic climate is mainly cold, it is far from being uniform. The Cenozoic period had seen constant volcanic activity in this continent, and it is possible to find steaming ground in some circumpolar islands, such as Deception Island [1].

**Fig. 1:**
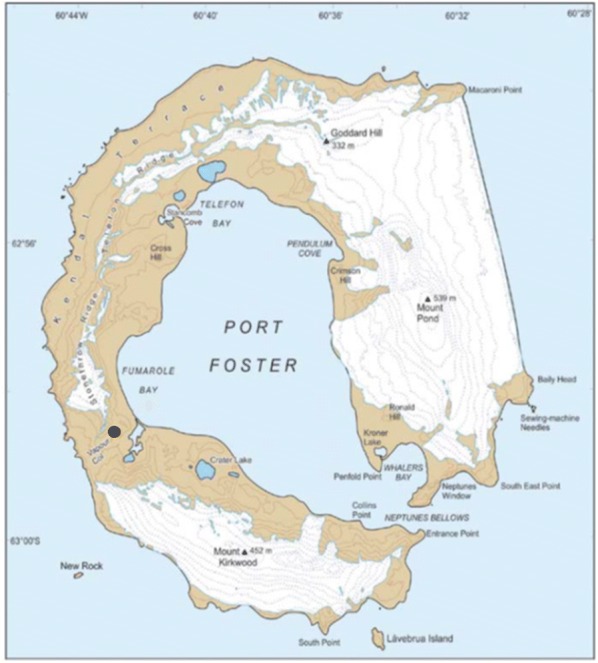
Map of Deception Island (South Shetland Islands, Antarctica). The black dot indicates the sampling site selected in the present work. Courtesy of British Antarctic Survey

Deception Island is one of the seven islands that constitute the South Shetland archipelago of the Antarctic continent (Fig. 1). This island is a stratovolcano with horseshoe-shaped due to the sinking of the central part of 17 km diameter. The volcano rises 1400 m from the seafloor to a maximum height of 540 m above sea level and lies on the expansion axis of the Bransfield rift, which separates the South Shetland Islands from the Antarctic Peninsula [2]. The age of this island is less than 780 Ka and was probably formed by the collapse of the upper part of an ancient composite volcano that probably became active. As a result of this volcanic activity, the island is composed mainly of andesite effusions, a pyroclastic rock. Although major volcanic eruptions occurred in 1967, 1969 and 1970, today the presence of geothermal volcanic activity is represented by fumaroles and hot soils [3].

The presence of a large number of fumaroles and other geothermal areas are characteristics that make the island interesting for the study of microorganisms, specifically thermophiles. One of the most predominant groups of bacteria found in geothermal soils in Antarctica belongs to the aerobic endospore forming *Bacillus*. So far, thermophilic bacteria found in these geothermal areas belong to the Bacillaceae family [1, 4].

Recently, studies using denaturing gradient gel electrophoresis (DGGE) using the 16S ribosomal gene were used to analyze bacterial diversity present in a soil sample taken from Fumarole Bay, Deception Island [5]. The study indicated the presence of bacteria from the genera *Geobacillus*, *Bacillus*, *Brevibacillus*, *Thermus* and uncultured sulphate reducing bacteria, some of them reported before in other Antarctic geothermal sites. Regarding Archaea, only few microorganisms have been described. These includes *Thermococcus* and *Pyrococcus* [6, 7].

Thermophiles and hyperthermophiles are source of novel enzymes which present biochemical characteristics that differ from their mesophilic counterparts. Therefore, they are currently being studied and have also been described from Antarctic microorganisms. These enzymes called thermoenzymes are focus of interest in both academic and industrial research, mainly due to their high thermal stability (resistance to inactivation at high temperatures) and optimal activity at high temperatures. These enzymes are adapted to function at growth conditions of the microorganism from where they come. The extreme temperature, pH, salinity, in many cases define the optimal conditions at which the enzymatic activity can be detected [8–10]. They also play an important role in the growing biotech market, with applications in agriculture, biomedicine and industry, among others, due to their thermal stability which facilitate its use in processes requiring high temperatures.

One of the enzymes of high scientific and applied interest is glutamate dehydrogenase (GDH). GDH plays a major role in the metabolism of carbon and nitrogen [11]. It belongs to the family of oxidoreductases and is widely distributed in Eukarya, Bacteria and Archaea. Its function is to reversibly catalyze oxidative deamination of glutamate to alpha-ketoglutarate and ammonium [10, 12]. In applications, oxidoreductases like GDH have been highly regarded for their specificity and ability to operate in photochemical and electrochemical systems as biosensors. However, their use has been limited due to the instability at high temperatures, in the presence of organic solvents and denaturing agents [13].

Many commercial kits for the quantification of ammonium and glutamate in biological fluids or food products are based on redox reaction of NAD(P)^+^ [14, 15].

In the food and wine industry, the amount of ammonia can be determined using GDH. The enzyme allows to determine the degree of decomposition of foods, quantifying the concentration of ammonia produced due to the bacterial degradation of proteins, peptides and amino acids [16]. However, currently the bovine GDH used in this kit lacks the stability required for its use at room temperature and for long term storage (Roche 1996–2010).

In this work, we report the isolation of a new thermophilic microorganism called PID15 from samples of Deception Island collected during the Antarctic Scientific Expedition ECA 46, which has a glutamate dehydrogenase able to work in a range of temperatures from 37 to 50 °C and at pH from neutral to slightly alkaline showing properties for potential biotechnological applications.

## Materials and methods

### Sample collection and culture conditions

Soil samples were collected during Antarctic Chilean Expedition 46 (ECA 46), from geothermal sites of “Cerro Caliente”, Deception Island (S62°58.045′, W60°42.609′), Antarctica. Temperature and pH of the selected area was measured. The temperature of the site range from 75 to 95 °C and pH was 5.5. All samples were aseptically collected and transferred to sterile vials.

2.0 g of environmental sample were inoculated in liquid trypticase soy broth and incubated at 50 and 70 °C. The mixed cultures obtained were passed to solid media containing 1.5% Gelrite (Merck & Co., Inc) and 0.75 g/L MgCl_2_, to increase polymerization capacity, and were incubated at the same temperatures already mentioned.

The microorganism isolated was obtained using the method of serial dilutions in liquid media combined with solid media culturing at temperature and pH optimum for the culture.

### Growth curve

PID15 isolate presented the higher GDH specific activity found among several cultures, measured by using a protocol for activity described in next sections. Then PID15 isolate was selected for its characterization. For optimum temperature, the microorganism was incubated during 22 h incubated at the range of temperatures between 20 and 80 °C. For optimum pH, measurements were performed in the range of 4.0–11, using different buffers at 25 mM (MES, HEPES, Tris–HCl and CAPS buffers). The optical density of the culture was measured at 600 nm by a spectrophotometer (Shimadzu). For the construction of the growth curve, 1 L of culture medium was inoculated at 10% with PID15 under optimal conditions of temperature and pH and 120 rpm of agitation. Growth was followed measuring the absorbance at 600 nm by a spectrophotometer during 33 h. Aliquots of 1 mL were taken each hour. Every 5 h 50 mL of culture were collected and was prepared the crude extract to measure of GDH activity.

### Morphological and biochemical characterization

Cell morphology was examined by scanning electron microscopy (SEM) and transmission electron microscopy (TEM) using an electron microscope JEOL JSM-T300 (resolution up to 10 nm) and a Philips Tecnai 12 Bio Twin TEM operating at 200 kV, respectively. Samples were washed with Tris–HCl buffer pH 7.0 in order to reduce the salt content. For TEM samples were fixed in 4% (v/v) formaldehyde. Gram staining was also performed. Biochemical characterization was performed using API20 E Kit (bioMérieux, Inc.) and this study was corroborated by using test tubes with commercial media: triple sugar Iron (TSI), lysine iron agar (LIA), ornithine indole motility (MIO), simmons citrate, urea, Hugh and Leifson (OF). Semisolid media were prepared for tests of carbohydrates oxidation and solid media was used for tests of gelatin and starch hydrolysis. All media were purchased from BD Biosciences.

### PCR amplification of 16S rRNA gene

Genomic DNA was extracted from PID15 using phenol cloroform method [17]. The gene 16S rRNA was amplified using universal primer 1492R (5′-TACCTTGTTACGACTT), specific primer for Archaea domain 21F (5′-TCCGGTTGATCCYGCCGG-3′) and specific primer 27F (5′-AGAGTTTGATCCTGGCTCAG-3′) for Bacteria respectively [18]. The reaction mix consisted of using 2.5 U of Taq DNA polymerase, 200 μM of each deoxy (d) nucleotide (dATP, dCTP, dGTP and dTTP), 1× of reaction buffer, 0.75 mM of MgCl_2_ and 0.5 µM of each primer. The following thermal conditions were applied: 95 °C for 45 s, 55 °C for 45 s, 72 °C for 45 s. Each cycle was repeated thirty times and a final elongation step of 72 °C for 10 min was added. Amplification reactions were carried out using a Palm Gradient Cycler (Corbett). Verification of PCR amplification was carried out by running the sample on a 1.5% agarose gel stained with SYBR gold (Invitrogen).

### Phylogenetic analysis

PCR product was sequenced using the set of primers described above, analyzed, and manually edited using ChromasPro software (Technelysium Pty Ltd.). Clustal W software was used to align the partial sequence of 16S rRNA gene from PID15 with selected sequences retrieved from GenBank. The software package MEGA4 [19] was used for a phylogenetic analysis and a tree was constructed using the Neighbor-Joining method [20]. Distances were computed using the maximum composite likelihood method with a bootstrap analysis of 1000. *Escherichia coli* JQ661175 was used as outgroup. The accession number of GenBank for the 16S rDNA gene of PID15 is JQ965669.

### Crude extract preparation and glutamate dehydrogenase activity

For crude extract preparation, 200 mL of cultures were centrifuged at 7300×*g* for 15 min and the cells obtained were resuspended in 1.5 mL of 50 mM Tris–HCl pH 8.0 containing 1 mg/mL lysozyme and incubated for 1 h at 37 °C. Subsequently, the samples were sonicated for 2 min in three different pulses in a Branson sonicator, 1510R-MT. Finally, the samples were centrifuged for 30 min at 81,650×*g* to separate the soluble crude extract from insoluble fraction using a Hitachi centrifuge (Himac CP80WX). GDH activity was measured spectrophotometrically by measuring the glutamate-dependent reduction of NAD^+^ at 340 nm at 37 and 50 °C shown by an increase of absorbance at 340 nm. One unit (U) of enzyme activity is defined as the amount of enzyme that catalyzes the formation of one µmol of NADH per minute. The reaction was carried out in a final volume of 1 mL containing 10 mM glutamate, 0.4 mM NAD^+^ and 100 mM EPPS pH 8.0. Protein concentration was estimated by Bradford method [21] using a Bio-Rad protein assay.

## Results and discussion

In this work it was possible to isolate 10 microorganisms from Scientific Expedition ECA 46, recollected from “Cerro Caliente”, Deception Island. Selected samples were obtained from sites with geothermal activity, with original temperatures optimal for the development of thermophilic microorganisms (above 50 °C). Crude extracts from those microorganisms were measured for GDH activity. The mayor activity for this enzyme was found in a microorganism called PID15.

PCR amplification was performed using universal primers for Bacteria and Archaea domain. All microorganisms studied belong to Bacteria domain.

**Fig. 2:**
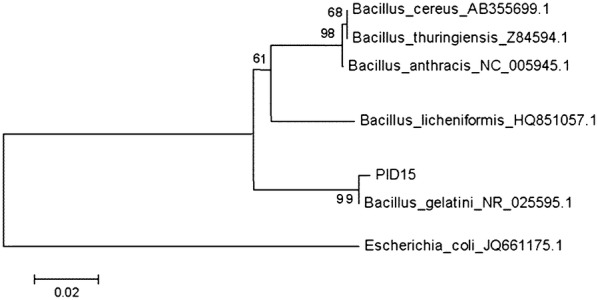
Phylogenetic position of PID15 16S rRNA gene. Phylogenetic tree was constructed using the Neighbor-Joining method with bootstrap of 1000

Phylogenetic relationship of 16S rRNA revealed that PID15 is closely related to *Bacillus gelatini* (Fig. 2). This microorganism was described as a contaminant in the gelatin production [22] and it has never been reported to be present in Antarctica before. Despite that the presence of thermophilic long rods from the genera *Bacillus*, *Geobacillus* and *Brevibacillus* has been previously described in Deception Island [5, 23]. The 16S rRNA from PID15 showed to have 99% identity with the 16S rRNA from *B. gelatini.* Nevertheless, biochemical tests showed several differences existing among them (see Table 1).

**Table 1:** Biochemical characterization of by API20 E kit (bioMérieux, Inc.) and confirmed by tube assay

Biochemical test	PID15	*B. gelatini* (DSM 15865)
Characteristics		
Gram	+	+
Temperature of growth (°C)	45–65	40–60
pH growth range	6.0–11.0	4.0–10.0
Motility	+	+
Aminoacid degradation		
l-Arginine dihydrolase	(−)	(−)
l-Lysine decarboxylase	+	(−)
l-Ornithine decarboxylase	+	(−)
Tryptophan deaminase	(−)	(−)
Indole	(−)	(−)
Metabolism		
Hugh-Leifson (oxidation)	+	+
Hugh-Leifson (fermentation)	(−)	(−)
Methyl red	(−)	(−)
Catalase	+	+
Oxidase	(−)	(−)
Voges Proskauer	(−)	(−)
Hydrolysis of		
Gelatinase	+	+
Starch	(−)	(−)
β-galactosidase	(−)	(−)
Carbon source		
Citrate	(−)	(−)
d-Glucose	+	V
d-Mannitol	+	V
Galactose	+	(−)
Ribose	+	V
d-Sucrose	+	(−)
Lactose	+	(−)
Others		
H_2_S production	(−)	(−)
Nitrate reduction	(−)	(−)
Urease	(−)	(−)

Strains: *B. gelatini* (DSM 15865) [22] and PID15 from the present study. Both microorganisms were tested under similar conditions. (+), positive; (−), negative; v, variable

**Fig. 3:**
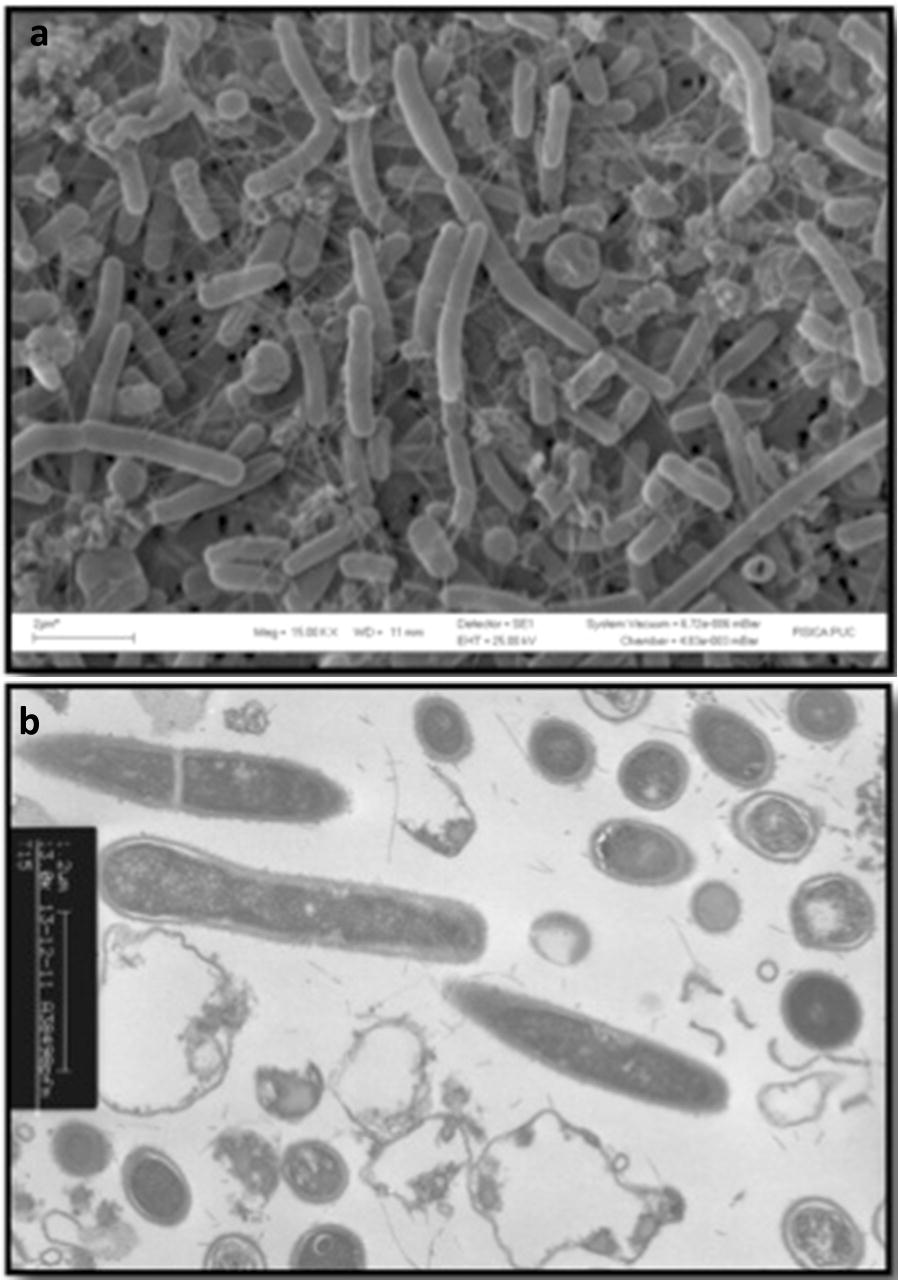
PID15 electron microscopy. **a** Scanning microscopy with ×5000 of magnification. **b** Microscopy of transmission and magnification of ×13,000

PID15 cells were Gram-positive long rods of 2.0–8.0 µm of size (Fig. 3a). Its colonies were beige, circular with irregular edges and with 5.0–10 mm of diameter after 1 day of incubation at 50 °C. Table 1 shows the comparison between PID15 and *B. gelatini* (DSM 15865). The temperature range for growth was very similar for both strains (45–65 °C and 40–60 °C for PID15 and *B. gelatini*, respectively), but the pH range for its activity was more neutral to alkaline for PDI15 (6.0–11) in comparison to *B. gelatini*, (4.0–10), showing an optimum growth at pH 8.0 and 50 °C for PID15 [24]. Doubling time (t_d_) for PID15 microorganism was 4.92 h under optimum conditions. Biochemical characterization of PID15 and *B. gelatini* presented several differences which indicates that PID15 could correspond to a new microorganism. However, additional studies must be performed.

PID15, showed the presence of l-lysine decarboxylase and l-ornithine decarboxylase activities. The microorganism was also capable to use galactose, d-sucrose and lactose as unique source of carbon.

The API20 E assay shown that PID15 has the following enzymatic activities: lipase (C8), esterase (C4), β-glucosidase, leucine arylamidase and cystine arylamidase. These activities are important from biotechnological point of view, since lipases and esterases, for example, are widely used for application in food, detergent, pharmaceutical, leather, textile, cosmetic and paper industries [25].

**Fig. 4:**
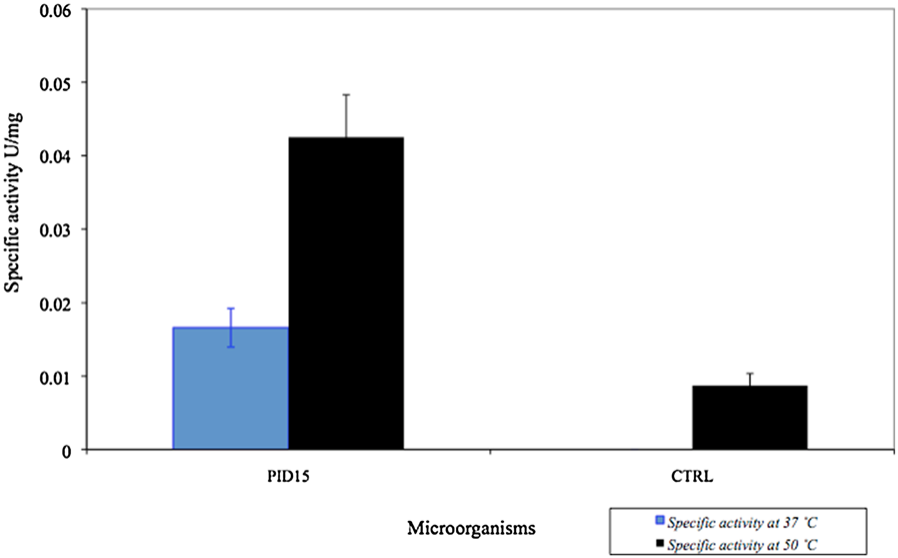
Screening of GDH specific activity at 37 °C and 50 °C. The specific activity was determined for the oxidative deamination reaction at 37 °C and 50 °C. As control glutamate dehydrogenase from GWE1 was used [26]. Error bars show the variation obtained from three biological replicates

**Fig. 5:**
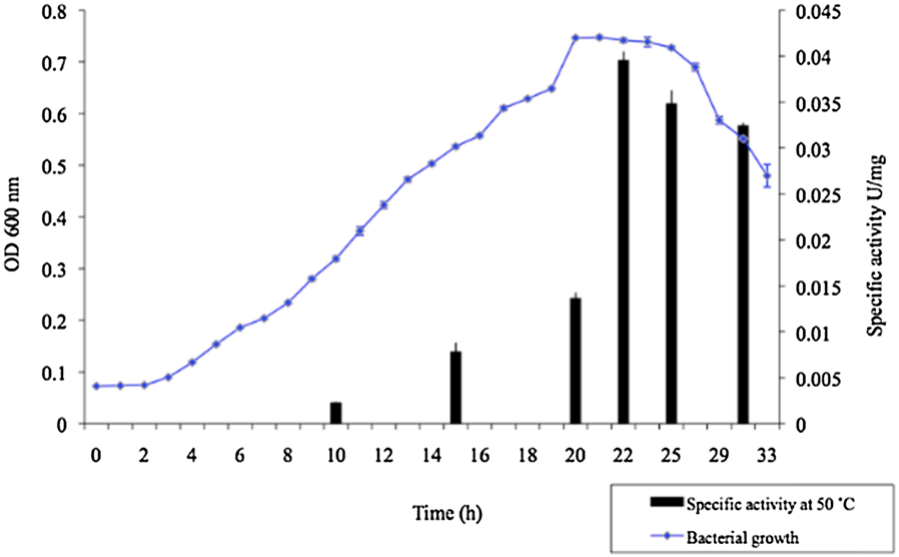
Bacterial growth curve and GDH specific activity. The specific activity was determined in the oxidative deamination reaction at 50 °C. Error bars show the variation obtained from three biological replicates

We focused our interest in GDH activity due to its promising biotechnological applications. We choose for the measurement of activity the direction of oxidative deamination reaction and NAD^+^ as coenzyme due to its higher thermal stability compared with NADP^+^ [26]. GDH activity was measured in PID15 isolate at two different temperatures 37 and 50 °C (Fig. 4), thinking in potential industrial applications. Specifically, 37 °C for use in clinical applications for the detection of ammonia in body fluids and 50 °C for the application in food industry. The PID15 GDH enzyme showed activity at both selected temperatures. This is important since a wide range of temperature defines a higher number of industrial processes where this enzyme could be used. The optimal specific activity of GDH from PID15 was at 50 °C and pH 8.0 [24]. The maximal specific activity was obtained at 22 h of microbial growth (Fig. 5). Therefore, GDH enzyme from PID15 represents a good candidate for further characterization, since it has a good activity at 50 °C, classifying it as a thermoenzyme.

## Conclusion

Our results indicate that Antarctic strain PID15 is a Gram positive microorganism that growth in a temperature range from 45 to 65 °C and a pH range from 6.0 to 11.0. Based on the phylogenetic analysis of the 16S rRNA gene, PID15 microorganism is closely related to *Bacillus gelatini.* Additionally, it has a glutamate dehydrogenase enzyme which can efficiently perform catalytic transformation for the oxidative deamination reaction at 37 °C and 50 °C, making this enzyme a potential candidate to be used in industry, for replacement of currently commercial GDH present in kits used for quantification of ammonium and glutamate in biological fluids or for applications in food products.

## Abbreviations

NAD(P)^+^: nicotinamide adenine dinucleotide phosphate
NAD: nicotinamide adenine dinucleotide
GDH: glutamate dehydrogenase
DGGE: denaturant gradient gel electrophoresis
ECA: Antarctic Scientific Expedition
HEPES: 4-(2-hydroxyethyl)-1-piperazine ethane sulfonic acid
CAPS: 3-(cyclohexylamino)-1-propanesulfonic acid
EPPS: 4-(2-hydroxyethyl)-1-piperazine propane sulfonic acid
SEM: scanning electron microscopy
TEM: transmission electron microscopy
